# Traumatic subgaleal hematoma drainage in an adolescent: a case report and review of the literature

**DOI:** 10.3389/fped.2023.1182899

**Published:** 2023-05-30

**Authors:** Blandine Aubert, Manon Cadoux, Cyril Sahyoun

**Affiliations:** Division of Pediatric Emergency Medicine, Children’s Hospital of Geneva, Geneva, Switzerland

**Keywords:** pediatric, drainage, head trauma, subgaleal hematoma, trauma

## Abstract

**Background:**

Subgaleal hematoma is a well-known life-threatening complication of instrumentation at birth. Even though most cases of subgaleal hematomas occur in the neonatal period, older children and adults are also at risk for subgaleal hematomas and their complications, following head trauma.

**Objective:**

We hereby report the case of a 14-year-old boy who presented with a traumatic subgaleal hematoma requiring drainage and review the relevant literature regarding potential complications and indications for surgical intervention.

**Results:**

Infection, airway compression, orbital compartment syndrome and anemia requiring transfusion are potential complications of subgaleal hematomas. Although rare, surgical drainage and embolization are occasionally required interventions.

**Conclusion:**

Subgaleal hematomas following head trauma can occur in children beyond the neonatal period. Large hematomas may require drainage to relieve pain or when compressive or infectious complications are suspected. Although usually not life-threatening, physicians taking care of children must be cognizant of this entity when caring for a patient with a large hematoma following head trauma and in severe cases, consider a multidisciplinary approach.

## Introduction

Subgaleal hematomas occurs when blood accumulates between the periosteum and the aponeurosis due to the tearing of emissary veins. It is a known complication of instrumentation at birth, and neonates can have up to 50% of the body blood volume accumulate in the space, leading to hemorrhagic shock and death (1, 2). Even though most cases of subgaleal hematomas occur in the neonatal period, older children and adults are also at-risk following head trauma. In this report, and we hereby report the case of a 14-year-old child who presented with a traumatic subgaleal hematoma with worsening headache and without hemodynamic instability, requiring drainage and review the available literature on the topic.

## Case description

A 14-year-old otherwise healthy boy was brought to the pediatric emergency department (PED) of a tertiary care urban medical center by his parents 3 days after sustaining a skateboarding injury. The patient reports falling directly on his head from his height, striking the top of his head on the halfpipe cement structure of the skatepark, while not wearing a helmet. He denies loss of consciousness, nausea, vomiting, abnormal behavior, or any other injury at the time and did not seek medical consultation. He has however since complained of a progressively worsening headache when moving his head from side to side and felt a lump on the frontoparietal part of his head. With the headache becoming severe, the patient and his family decided to seek care in the PED.

Vital signs revealed a normal heart rate of 80 beats per min, a slightly elevated blood pressure of 133/90 mmHg, a normal respiratory rate of 16 per min, normal oxygen saturation at 98% in ambient air, and no fever. The patient appeared well, was alert, interactive and conversive, he complained of mild discomfort at rest but pain that is difficult to tolerate when ambulating, particularly when moving his head from side to side. On inspection, a large area of fluctuant swelling on the right frontoparietal region of the scalp was noted, which was painful to palpation. The remainder of the examination including the neurological examination showed no abnormalities.

## Diagnostic assessment

**Figure 1:**
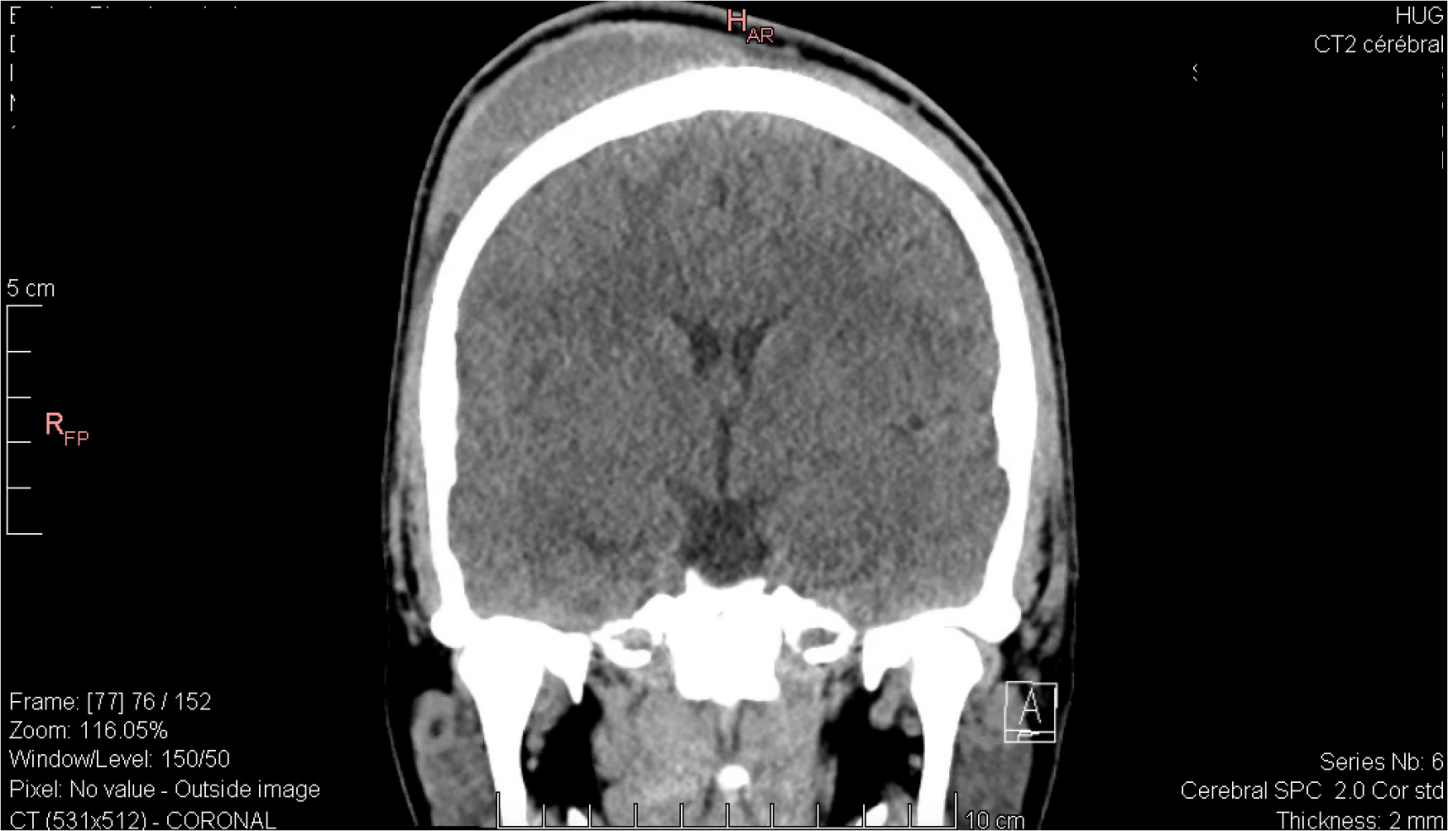
Coronal view of the head computed tomography scan revealing a large right-sided subgaleal hematoma.

Given the suspicion for an underlying skull fracture and given the severity of the headache, the decision was made to obtain a non-contrast computed tomography (CT) of the head. CT revealed no evidence of intracranial hemorrhage, intracranial hypertension, or skull fracture, and confirmed the diagnosis of a right voluminous frontoparietal subgaleal hematoma extending to the left frontal region (Figure 1).

Laboratory examination showed normal coagulation studies and no thrombocytopenia. Neurosurgical consultation was obtained to help guide management, and given the severity of the headache, the decision was made to drain the collection. Bedside needle drainage was performed by the neurosurgeon in the PED, with insertion of a butterfly needle in sterile conditions and aspiration of 100 cc of blood, followed by application of a compression bandage. The patient was subsequently discharged from the PED with a follow up appointment with his pediatrician.

The compression bandage was kept for 48 h, and headaches resolved in that timeframe. There were no other symptoms in the days and weeks following his admission to the PED. No further analgesia was needed, and the swelling progressively decreased then completely resolved over the course of 3 weeks, as documented by a telephone conversation with the patient and his family.

## Discussion

Subgaleal hematoma is a complication known to pediatricians attending the delivery room. The condition is much rarer in older children. The patient described above presented with severe pain from a traumatic subgaleal hematoma, improved with needle drainage, and had no further complications.

We searched PubMed for articles published in the last forty years in English with the title containing “subgaleal hematoma,” with available abstracts and accessible full-texts, and retained only the ones involving otherwise healthy children outside of the neonatal period. Our literature review reveals that the main reasons for subgaleal hematomas in children are minor head injury, hair pulling or braiding and trauma leading to a skull fracture. Reports of child abuse with hair pulling, hair braiding or corporal punishment leading to subgaleal hematoma are also described (Table 1).

**Table 1:** Literature review and outcome summary of relevant publications related to pediatric subgaleal hematoma.

Author, year of publication	Number of patients (age)	Mechanism and complications	Known coagulopathy	Treatment	Outcome (time of follow up when known)
Pope-Pegram and Hamill (3)	1 (7 Y)	Head strike on a dresser. Subgaleal hematoma and secondary superior orbit subperiosteal hematoma with increased intraocular pressure	None	Surgical drainage	Uneventful
Nichter et al. (4)	1 (12 Y)	Subgaleal hematoma following mild head trauma leading to airway obstruction	None	Surgical incision drainage Tracheostomy	Hypopigmentation of the forehead (12 mo)
Lee et al. (5)	1 (13 Y)	Left parietal region strike leading to subgaleal hematoma and secondary proptosis	None	Surgical drainage	Uneventful (1 mo)
Cooling and Viccellio (6)	1 (23 mo)	Subgaleal hematoma following minor head trauma	None	None	Uneventful
Pomeranz et al. (7)	1 (6 Y)	Roller-skating fall	None	Surgical drainage after 11 days because of increasing pain and proptosis	Unknown
Vu et al. (8)	1 (8 Y)	Hair pulling causing subgaleal hematoma	None	None	Uneventful (5.5 weeks)
Raffini and Tsarouhas (9)	1 (17 Y)	Hair pulling causing subgaleal hematoma	Von Willebrand Disease diagnosed after presentation	Drainage in the emergency department	Re-accumulation of fluid 72 h after first drainage.
Fujisawa et al. (10)	1 (12 Y)	Subgaleal hematoma following hair pulling with secondary extension into the orbit	None	Needle drainage and compression bandage followed by surgical drainage	Uneventful (29 days)
Seifert and Puschel (11)	1 (3 Y)	Hair pulling in a context of child abuse, extensive subgaleal hematoma	Unknown	Unknown	Unknown
Sillero Rde (12)	1 (14 Y)	Bicycle fall, massive subgaleal hematoma, otorrhagia	None	Surgical incision drainage	Uneventful
Karcioglu et al. (13)	1 (8 Y)	Subgaleal hematoma following mild head trauma with secondary intraorbital hemorrhage and ocular compromise	None	Intraorbital drainage and midcoronal incision	Improvement of visual abilities (3 weeks)
Kim and Taragin (14)	1 (9 Y)	Hair pulling and hair braiding	Factor XIII deficiency	Surgical drainage	Unknown
Onyeama et al. (15)	1 (31 mo)	Hair braiding	None	None	Uneventful (resolution in 2 weeks)
Koizumi et al. (16)	1 (15 Y)	Subgaleal hematoma from a branch of the right superficial temporal artery	None	Percutaneous hematoma aspiration and endovascular embolization	Uneventful (1 year)
Natarajan et al. (17)	1 (6 Y)	Minor head injury	Factor XIII deficiency	Surgical drainage	Uneventful (3 mo)
Stalder et al. (18)	1 (25 mo)	Minor head trauma in a child	Kasabach-Merrit phenomenon	Secondary surgical drainage and debridement after failure of conservative treatment	Uneventful (1 Y)
Kichari and Gielkens (19)	1 (15 Y)	Minor trauma	None	Compression bandage	Uneventful
Yamada et al. (20)	1 (6 Y)	Bicycle fall, skull fracture with massive subgaleal hematoma and epidural hematoma	None	Secondary surgical drainage after failure of conservative treatment	Uneventful
Barry et al. (1)	1 (8 mo)	Minor head trauma, linear head fracture, infection	None	Surgical drainage via needle aspiration	Uneventful (3 mo)
Shamji and Jacoby (21)	1 (2 Y)	Chronic hair traction in a context of child abuse	None	Surgical drainage	Unknown
Edmondson et al. (22)	1 (16 Y)	Traumatic hair pulling	None	Surgical due to extension with periorbital swelling	Uneventful (3 mo)
Wajima et al. (23)	1 (14 Y)	Spontaneous subgaleal hematoma of unknown origin	None	Vessel embolization due to conservative treatment failure	Uneventful (1 Y)
Chen et al. (24)	1 (39 Y)	Hair twisting into air compressor, scalp laceration and subgaleal hematoma	None	Conservative: compression bandage	Uneventful (3 mo)
Jenkins et al. (25)	1 (10 Y)	Struck by football, subgaleal and secondary subperiosteal hematoma of the orbit	Heterozygous form of Factor VII deficiency	Surgical incision drainage	Uneventful
Scheier et al. (2)	1 (10 Y)	Non abusive hair pulling, worsening anemia and fever	None	Surgical drainage. Blood transfusion	Uneventful
Puri et al. (26)	1 (13 Y)	Hair pulling, orbital subperiosteal hematoma causing vision loss	Under investigation at time of publication	Surgical incision drainage	Uneventful at hospital discharge
Bowens and Liker (27)	1 (4 Y)	Child abuse (corporal punishment)	None	None	Unknown

Y, years; mo, months.

Intervening on a subgaleal hematoma is rarely needed in children. The symptoms of pain and swelling usually resolve within a few days to weeks after the injury and drainage is rarely necessary. The usual work up performed includes complete blood count looking for thrombocytopenia and coagulation profiles looking for a bleeding diathesis (9, 14, 17, 25).

We hereby discuss the potential complications of traumatic subgaleal hematomas and related interventions.

## Possible complications

### Airway compression

Airway compression is a rare complication of a traumatic subgaleal hematoma. Nichter et al. describe a 12-year-old healthy child who suffered a mild blow to the head leading to a massive hematoma of the head and neck resulting in airway compression. Emergency tracheostomy and surgical drainage was required, with concern for impending skin necrosis (4). In their report, the mechanism leading to the massive swelling remained unclear, but the hypothesis was that the attachment of the galea to the zygomatic arch can be delicate and a large hematoma could cross this boundary. The patient described in their report had a favorable outcome at twelve months follow up with only spotty hypopigmentation in the forehead region.

### Orbital compartment syndrome

Orbital compartment syndrome has also been described as a complication of subgaleal hematoma, with extension of the hematoma to the orbital subperiosteum (3, 5, 7, 10, 13, 19, 25, 26). Patients usually present with a history of head trauma and progressive exophthalmia, blurred vision, ptosis, and pain. In such cases, prompt surgical drainage of the hematoma may be required, as blindness secondary to increased intraocular pressure is a possible outcome of untreated orbital compartment syndrome.

### Infection

Infection of subgaleal hematomas secondary to a scalp laceration during instrumented delivery has been well described in the neonatal period (1). In older children, subgaleal hematomas can also get infected through local spread of bacteria, but hematogenous dissemination during an upper respiratory infection has also been reported: Barry et al. report a healthy 8-month-old girl who presented with an infected subgaleal hematoma following a minor fall. Physical examination revealed no scalp laceration, yet cultures of the hematoma grew Streptococcus pneumoniae. The origin of the infection was hypothesized to be hematogenous spread of S. pneumoniae colonizing the nasopharynx, through one of the anastomoses of the facial vascular system. The patient underwent four weeks of intravenous antibiotics regimen before hospital discharge. In the articles reviewed in this study, no prophylactic antibiotics were used after drainage in non-infected subgaleal hematoma.

### Need for transfusion

It is worth noting that even though the relative volume of blood in traumatic hematomas is usually less important than in newborns, transfusion might be needed in some cases (2). In addition to the case reported by Nichter et al. and mentioned above where a transfusion was required after a liter of blood was drained from the patient's subgaleal hematoma, Scheier et al. also describe a case requiring a blood transfusion; a 10-year-old girl presented with a subgaleal hematoma following non accidental hair pulling. The patient underwent surgical drainage (250 ml of blood), and a transfusion was required for a hemoglobin nadir of 7.1 g/L, associated with tachycardia and fatigue. The outpatient follow-up showed no coagulopathy and no recurrence of the hematoma.

## Intervening on subgaleal hematomas

### Drainage

When the above complications are suspected, surgical drainage may be necessary. Drainage however carries a risk of complications, particularly iatrogenic infection, as emissary veins communicate with the dural sinuses.

In the patient described in this case report, drainage was deemed appropriate to relieve the discomfort of the patient, after weighing the risks and benefits of the procedure.

Subgaleal hematomas might also lead to calcification, motivating drainage to avoid such complications. In rare cases, calcifications can indeed lead to cranial deformation requiring reconstructive surgery, especially in patients with hematologic disease such as Kasabach-Merrit phenomenon, whereby a vascular lesion causes platelet and coagulation factor consumption (18). Concern for potential development of calcifications is however not a criterion for drainage, as calcifications are not prevalent, particularly in patients with no underlying disease. The decision to surgically treat hence depends on patient risk factors, symptoms intensity and evolution, and complications. A similar approach is also described in adult studies and reports (24).

When calcifications or complications are present, however, surgical drainage and evacuation may be warranted. Edmondson et al. report a case of a 16-year-old boy who presented with a large subgaleal hematoma following hair braiding 10 days prior, with magnetic resonance imaging revealing areas of calcification (22). There were no arguments for an underlying vascular malformation, however, because of hematoma extension to the periorbital space, the patient underwent drainage of calcified clots via multiple small incisions.

### Embolization

Embolization has also been described after subgaleal hematoma (16, 23). Wajima et al. report an otherwise healthy adolescent presenting to the emergency department with two days of headache and no recall of head injury. Swelling of the scalp was noted and a head CT confirmed a subgaleal hematoma. He was drained on initial presentation and returned two weeks later with further scalp swelling and pain. Because of the recurrence of the hematoma, the patient underwent embolization. The outcome was favorable with no recurrence of the hematoma.

Although rare, physicians must be cognizant of the complications above and the indications for intervention, when presented with a subgaleal hematoma in a pediatric patient.

## Conclusion

Although rare, subgaleal hematomas can occur in children beyond the neonatal period and are mostly associated with trauma, at times as benign as a minor fall, hair-braiding or hair pulling. While it is rare, voluminous subgaleal hematomas may require drainage to relieve pain or when compressive or infectious complications are suspected, especially in a patient with a known coagulopathy. Although usually not life-threatening unlike in the neonatal period, physicians taking care of children must be cognizant of this entity when caring for a patient with a large hematoma following head trauma, anticipate potential complications and consider a multidisciplinary approach with neurosurgery and hematology consultation if the presentation or evolution is atypical.

## Data Availability

The original contributions presented in the study are included in the article, further inquiries can be directed to the corresponding author.
